# Hepatitis B virus X protein in liver tumor microenvironment

**DOI:** 10.1007/s13277-016-5406-2

**Published:** 2016-09-23

**Authors:** Sha Fu, Rong-rong Zhou, Ning Li, Yan Huang, Xue-Gong Fan

**Affiliations:** 1 Department of Infectious Diseases, Key Laboratory of Viral Hepatitis of Hunan Province, Xiangya Hospital, Central South University, P. O. Box: 410008, Changsha, China; 2 Department of Blood Transfusion, Xiangya Hospital, Central South University, Changsha, China

**Keywords:** Hepatitis B virus, Hepatitis B virus X protein, Hepatocellular carcinoma, Tumor microenvironment, Inflammation

## Abstract

Encoded by the hepatitis B virus, hepatitis B virus X protein (HBx) is a multifunctional, potentially oncogenic protein that acts primarily during the progression from chronic hepatitis B to cirrhosis and hepatocellular carcinoma (HCC). In recent decades, it has been established that chronic inflammation generates a tumor-supporting microenvironment. HCC is a typical chronic inflammation-related cancer, and inflammation is the main risk factor for HCC progression. The viral transactivator HBx plays a pivotal role in the initiation and maintenance of hepatic inflammatory processes through interactions with components of the tumor microenvironment including tumor cells and the surrounding peritumoral stroma. The complex interactions between HBx and this microenvironment are thought to regulate tumor growth, progression, invasion, metastasis, and angiogenesis. In this review, we have summarized the current evidence evaluating the function of HBx and its contribution to the inflammatory liver tumor microenvironment.

## Introduction

Hepatocellular carcinoma (HCC) is one of the main causes of cancer-related deaths worldwide; every year, nearly 750,000 people are diagnosed with HCC [1]. A growing body of evidence has shown that chronic hepatitis B virus (HBV) infection is strongly associated with HCC development and that more than 50 % of HCC cases may be caused by persistent HBV infection [2]. In recent decades, extensive research has been conducted to elucidate the molecular events underlying HCC development and invasion; however, the carcinogenic mechanisms involved in this process remain unclear. Several mechanisms for HBV-induced hepatocarcinogenesis have been proposed including integration of HBV DNA into the host genome, long-term chronic liver inflammation, and carcinogenic effects of HBV antigens [3, 4]. Hepatitis B virus X protein (HBx), a multifunctional viral protein encoded by HBV, is considered to be one of the most important determinants of the pathology of HBV-related HCC (HBV-HCC). An accumulating body of evidence has suggested that HBx exerts its carcinogenic effects by activating a variety of cellular signaling pathways thereby controlling the cell cycle, proliferation, and apoptosis [5–7].

Tumor microenvironments generally comprise tumor cells, inflammatory cells, inflammatory cytokines, and other cellular and non-cellular components. Previous studies have suggested that tumor microenvironments play a crucial role in tumor initiation, invasion, and metastasis, via mechanisms including the generation of hypoxia, an alteration of microRNA (miRNA) expression profiles, and an increased adoption of stem cell phenotypes [8–10]. Inflammatory cells and signaling molecules constitute a large proportion of the tumor microenvironment; hence, it is also termed the “inflammatory tumor microenvironment” [11, 12]. The relationship between inflammation and cancer was first proposed in the nineteenth century. Subsequent studies have revealed that inflammatory diseases are associated with an increased risk of tumor development [13]. The inflammatory mediators are present in most tumor microenvironments, and an overexpression of inflammatory cytokines contributes to tumor development. Moreover, inhibition of inflammatory cytokines can reduce tumor invasion and progression [14]. As a result, inflammation is now recognized as one of the six hallmarks of tumor development and invasion [15]. HCC is a typical inflammation-associated tumor. All patients with HBV-HCC have an underlying chronic inflammatory liver disease caused by chronic viral infection, and HBx is thought to play a vital role in the regulation of chronic liver inflammation. Understanding the function(s) of HBx is fundamental to elucidating the mechanisms that underlie the generation and maintenance of the inflammatory tumor microenvironment during the development and progression of HBV-HCC. In this review, we focus on HBx and its contribution to the inflammatory liver tumor microenvironment in HBV-HCC. Molecules interacting with HBx in the liver tumor microenvironment are summarized in Table 1.

**Table 1:** Summary list of which HBx is involved in liver tumor microenvironment

Components	Effects	Study type	REFERENCES
Tumor cells	1. Promoting tumor cell proliferation	In vitro studies of transfected cell lines (HepG2) using JetPEI reagent	Cho et al. [7]
	2. Inhibiting apoptosis	In vitro studies of transfected cell lines HepG2 and HepG2.2.15 cells	Liu et al. [6]
	3. Inducing autophagy	In vitro studies of transfected cell lines human conjunctival epithelial Chang cells using Lipofectamine 2000 transfection reagent	Zhang et al. [16]
	4. Accelerating cell cycle progression.	In vitro studies of transfected cell lines Hep3B, Huh7, and HepG2 using CaPO_4_ precipitation method	Park et al. [5]
Immune cells	1. Promoting the apoptosis of CD8 + T lymphocytes	In vitro studies of isolated primary hepatocytes using	Lee et al. [17]
		recombinant baculovirus infection	
	2. Decreasing the generation of IFN-γ		
	3. Upregulation of major histocompatibility complex, ICAM-1,	In vitro studies of transfected cell lines HepG2 and UP74 cells	Zhou et al. [18]
	and Fas ligand	In vitro studies of transfected cell lines HepG2 and Huh7 cells	Kim et al. [19]
Hepatic stellate cells	1. Promoting hepatic stellate cell activation	In vitro studies of transfected cell lines hepatocyte cell lines Chang liver and HepG2 cells	Martin et al. [20]
	2. Promoting extracellular matrix remodeling, fibrosis		
	angiogenesis, HCC invasion, and metastasis		
	3. Promoting the proliferation of hepatic stellate cells	In vitro studies of transfected cell lines HepG2 and LX-2 using FuGENE HD transfection reagent	Bai et al. [21]
TGF-β	1. Upregulating TGF-β in a paracrine-dependent manner	In vitro studies of transfected cell lines hepatocyte cell lines Chang liver and HepG2 cells	Martin et al. [20]
	2. Participation in hepatic stellate cells activation	In vitro studies of transfected cell lines HL-7702 and L02 cells using a lipid-mediated method	Chen et al. [22]
	3. Transforming intrahepatic TGF-β signaling pathway from tumor-suppressive pSmad3C to tumor-supportive pSmad3L	In vivo studies of transgenic CD-1 mice and clinical specimens	Murata et al. [23]
	4. Cooperating with stem cell pathways to induce EMT	In vitro studies of transfected cell lines HMLE cell	Scheel et al. [24]
Interleukin family	1. Stimulating the production of IL-6 to mediate HCC development	In vitro studies of transfected cell lines L02 and SMMC-7721	Xiang et al. [25]
	2. Upregulating the expression of IL-8 to promote tumor growth and the malignant transformation of hepatocytes	In vitro studies of transfected cell lines SMMC-7721 and HepG2 cells	Wang et al. [26]
	3. Regulating other pro-inflammatory cytokines such as IL-18, IL-23, and TNF-α to induce liver chronic inflammation	In vivo studies of clinical specimens and in vitro studies of transfected cell lines HepG2 and Huh-7 cells	Xia et al. [27]
TNF-α	1. Upregulating TNF-α levels at transcriptional level	In vitro studies of transfected cell lines CCL13 and HepG2 cells	Lara-Pezzi et al. [28]
	2. Promoting tumor development and angiogenesis		Review [29]
COX-2	1. Upregulating the expression of MT1-MMP in a COX-2-dependent manner	In vitro studies of transfected cell lines CCL13 and HepG2.2.15 cell	Lara-Pezzi et al. [30]
	2. Exerting its anti-apoptotic effects by activating the COX-2/PGE(2) signaling pathway	In vitro studies of transfected cell lines Hep3B cell	Cheng et al. [31]
	3. Promoting tumor growth, invasion and metastasis	In vivo studies of clinical specimens and in vitro studies of transfected cell lines HepG2 cell	Liu et al. [32]
HIF-1α	1. Preventing HIF-1α degradation	In vitro studies of transfected cell lines CCL 13, HepG2, etc.	Yoo et al. [33, 34]
	2. Upregulating the expression of HIF-1α	In vitro studies of transfected cell lines Chang X-34 cells, HepG2, etc.	Yoo et al. [35]
Exosome	1. Negatively regulating the expression of exosomal miR-122	In vitro studies of transfected cell lines HepG2 and Huh-7 cell	Song et al. [36]
	2. Significantly altering the exosomal protein content	In vitro studies of transfected cell lines Huh-7 cell	Zhao et al. [37]

## Liver tumor microenvironment

The liver tumor microenvironment is an important factor in the development of tumor cells and in the regulation of tumor angiogenesis, invasion, and metastasis. It is broadly divided into its cellular and non-cellular components: the former includes liver cancer cells, hepatic stellate cells, fibroblasts, endothelial cells, mesenchymal stem cells, and immune cells; the latter includes inflammatory cytokines, growth factors, and extracellular matrix.

Previous studies have shown that epithelial cells can secrete some cytokines and recruit inflammatory cells to the tumor following oncogene activation or tumor suppressor gene inactivation [38]. In addition, tumor cells can upregulate the expression of proteases, cytokines, chemokines, and other inflammatory mediators, creating an inflammatory microenvironment that favors tumor survival. In other words, tumor cells can trigger an endogenous tumor-associated inflammatory response. Interactions between tumor cells and the matrix components of the inflammatory microenvironment regulate tumor survival, proliferation, invasion, and metastasis. Activated hepatic stellate cells reportedly participate in a wide range of physiologic and pathologic processes during the progression from chronic liver inflammation to cirrhosis and liver cancer [39, 40].

The roles of inflammatory cytokines, which are the main signaling molecules in the tumor microenvironment, in HCC development are unclear. A growing number of in vivo and in vitro studies suggest that the levels of inflammatory factors present in tumor tissues and serum, including interleukin (IL)-6, tumor necrosis factor-α (TNF-α), IL-1β, IL-10, and transforming growth factor-β (TGF-β), are often higher in patients with HCC. These inflammatory mediators may facilitate tumor growth, inhibit apoptosis, induce epithelial–mesenchymal transition (EMT), and promote tumor invasion and metastasis [41–44].

The immune status of the liver tumor microenvironment may also influence tumor progression and invasion. The prognosis of patients with an immunosuppressive signature in their tumor microenvironment is often poor, whereas patients with tumor-infiltrating lymphocytes have a reduced risk of recurrence after liver transplantation [45]. The HCC microenvironment also promotes immune escape, immune suppression, or both by accumulating immune-suppressive cells [46].

HBV-HCC is a common inflammation-related tumor. As a result, in most cases, HCC arises on cirrhotic livers further supporting the notion that chronic HBV infection plays a pivotal role in triggering and maintaining an inflammatory tumor microenvironment. This is mainly caused by HBV-related proteins, especially HBx, which drive chronic liver inflammation and stimulate the host immune response by triggering both common and etiology-specific signaling pathways.

## Properties of hepatitis B virus X protein

### Structure and hepatocellular carcinoma-related function of hepatitis B virus X protein

**Fig. 1:**
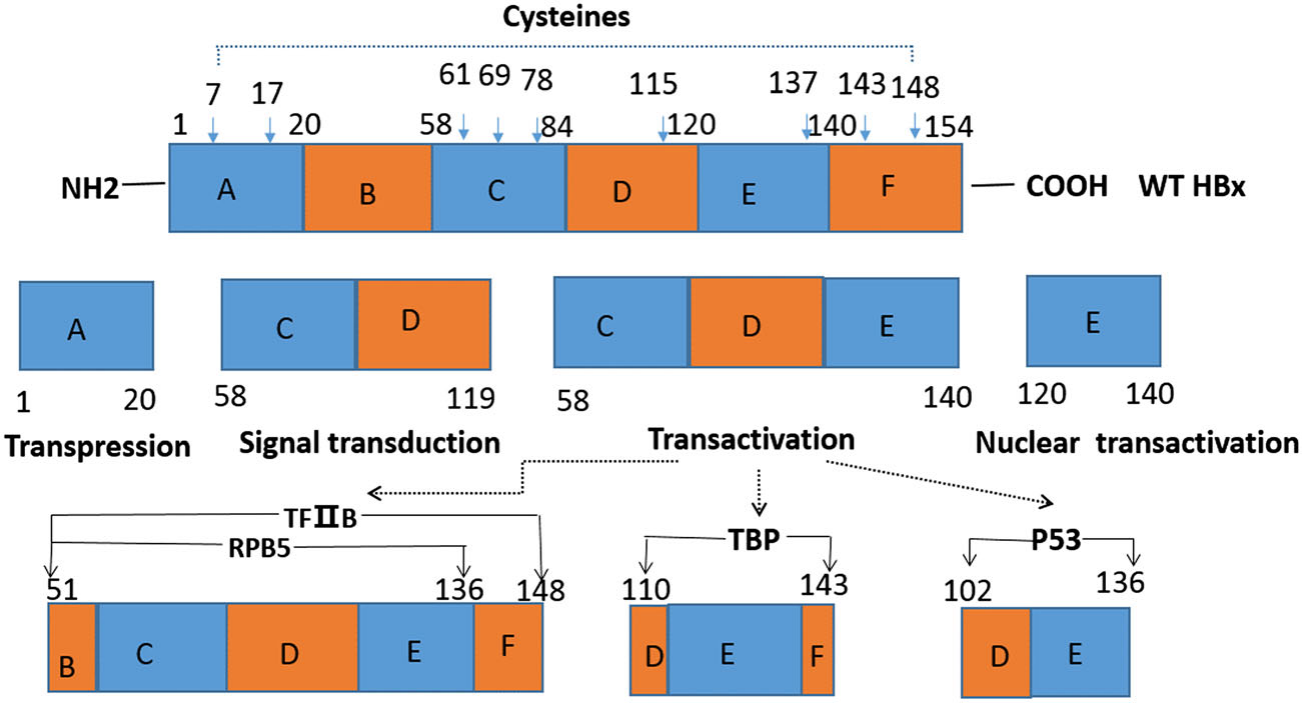
Domain structure of HBx with some possible functions. *WT* wild type. The functional domains of 154-aa HBx protein shown include the transpression domain (aa 1–20), the signal transduction domain (aa 58–119), the transactivation domain (aa 58–140), and the nuclear transactivation domain (aa120–140). Nine conserved cysteine residues that have proven to be crucial for HBx’s various functions are indicated by the *arrows* (aa 7, 17, 61, 69, 78, 115, 137, 143, and 148) [47]. HBx domains for interaction with various transcription factors such as *TFIIB* (aa 51–148), *RPB5* (aa 51–136), *TBP* (aa 110–143), *P53* (aa 102–136), etc. are shown [48–50]

There are four identified open reading frames (ORFs) of HBV, and the HBx-coding region has been described as the fourth ORF. HBx consists of 154 amino acids (∼16.5 kDa) and has six functional domains (A–F) that exhibit multiple functions including gene transactivation, transrepression, and cell signaling (Fig. 1). Nine conserved cysteine residues have been identified that are crucial for HBx function. These conserved cysteines have a distribution of two each in regions A and F, three in region C, and one each in regions D and E [47] (Fig. 1).

Over the past decade, efforts have centered on elucidating the role of HBx in the pathologic process of chronic HBV infection and HCC formation. Accumulated experimental evidence indicates that HBx is a multifunctional regulatory protein that exerts a pleiotropic effect on common biochemical pathways by communicating directly or indirectly with a range of host targets. This leads to pathologic processes such as viral propagation, gene transcription, signal transduction, and protein degradation that are ultimately associated with the development of HCC. The transactivation function of HBx was first postulated in 1981, and HBx has been characterized as a broad-spectrum activator of transcription with the ability to regulate all three classes of promoter [51–53]. HBx is able to induce growth factors, dysregulate the host’s immune response, activate cell survival signaling, and increase tumor cell invasion and metastasis.

### Subcellular localization of hepatitis B virus X protein

**Fig. 2:**
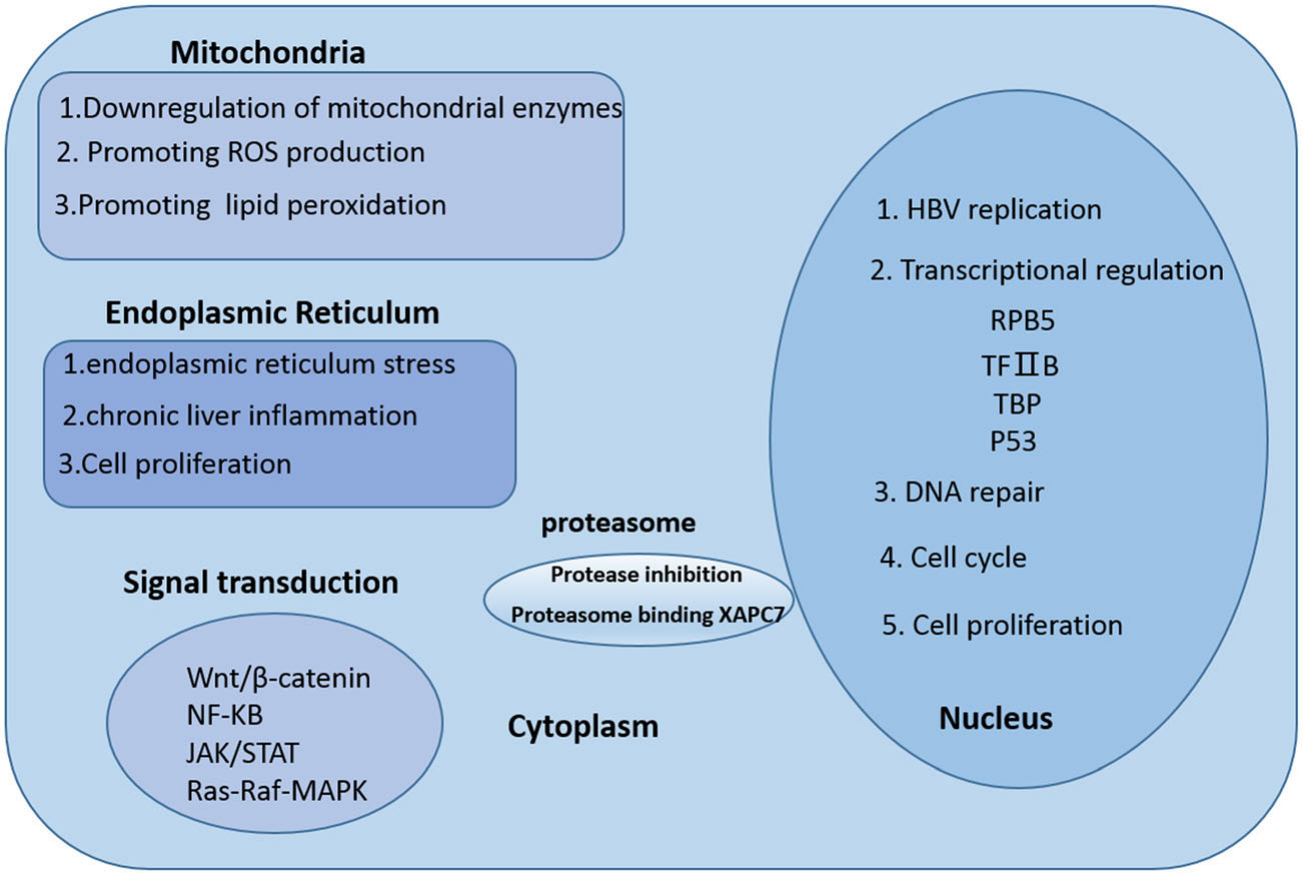
Subcellular localization of HBx and its various functions

The various biological functions of HBx occur in different intracellular locations, and this is critical for the pleiotropic effects of HBx in the liver tumor microenvironment (Fig. 2). HBx localized to the cytoplasm has been shown to participate in various cellular signal-transduction pathways related to development, invasion, migration, and recurrence of HCC, including the Wnt/β-catenin, nuclear factor κ-light-chain enhancer of activated B cells (NF-κB), Janus kinase/signal transducer and activator of transcription (STAT), and Ras/Raf/mitogen-activated protein kinase (MAPK) pathways [54–57]. In the nucleus, HBx is likely associated with gene regulation and HBV replication, which are both essential for chronic HBV infection. HBx can bind directly to transcription factors and activate gene transcription causing dysregulation of cell-cycle checkpoint controls, proliferation, apoptosis, and DNA repair [5, 6, 58]. In the mitochondria, HBx disrupts mitochondrial stability by downregulating mitochondrial enzymes and promoting reactive oxygen species (ROS) production and lipid peroxidation [59, 60]. These changes may explain the abnormal energy metabolism of tumor cells, the increase of ROS in the tumor microenvironment, the invasion and metastasis of tumor cells, and the resistance to cell death evident in HBV-HCC. Within the endoplasmic reticulum, HBx maintains chronic liver inflammation and proliferation by inducing endoplasmic reticulum stress [7, 61]. Reportedly, HBx also colocalizes with the proteasome and interacts specifically with a novel subunit of the proteasome complex (XAPC7) that is involved in protein degradation; HBx is thought to exert its various functions through the regulation of this process [62, 63].

### Roles of hepatitis B virus X protein truncation mutants in hepatocellular carcinoma

The HBx gene is easy to mutate and integrate into hepatocytes [64, 65]. Of all mutations of HBx, including point, insertion, and truncation mutations, deletion of the COOH terminal of HBx has attracted the most attention. Previous studies have indicated that COOH-terminal truncations of HBx play a significant role in the pathogenesis of HCC and that this mutation is often found in samples of HCC [66]. This truncated version of the HBx gene may encode a functional protein that is still capable of promoting malignant transformation. Several studies have reported that a natural mutant of HBx may induce the growth and proliferation of both healthy liver and human hepatoma cells [67, 68]. However, it is difficult to determine whether these mutants are drivers of carcinogenesis and how these structural mutations affect the functional behavior of HBx.

## Hepatitis B virus X protein and components of the liver tumor microenvironment

### Hepatitis B virus X protein and tumor cells

The multifunctional viral regulator HBx is often highly expressed in the tumors of patients with HBV-HCC, and it is considered to be a key determinant of the pathogenesis and carcinogenesis of HBV-related liver diseases. HBx regulates the biological behavior of tumor cells through its transcriptional regulation and transactivation activities. Reportedly, HBx promotes hepatoma cell proliferation via the activation of cellular signaling pathways, dysregulation of cell-cycle checkpoint controls, and regulation of non-coding RNAs and transcription factors [5, 7, 69]. The impact of HBx expression on cellular apoptosis has also been analyzed. According to previous studies, HBx regulates apoptosis by acting on caspases, the mitochondria, and SIRT. Although HBx exerts varying effects on apoptotic pathways in different model systems, the modulation of apoptotic pathways by HBx is considered to be closely related to the development of HBV-HCC [6]. Recently, it was suggested that HBx is a major contributor to the induction of autophagy during HBV infection. Zhang et al. [16] demonstrated that HBx dephosphorylates and activates DAPK, thus inducing autophagy in a Beclin 1-dependent manner. Moreover, HBx triggers malignant transformation in the pathogenesis of HCC by promoting properties characteristic of cancer stem cells [70]. Through these mechanisms, HBx is believed to contribute to hepatocarcinogenesis.

### Hepatitis B virus X protein and immune cells

Once patients are infected with HBV, the development of liver disease and the outcome of the disease are largely determined by immune-mediated host–virus interactions and particularly by the cellular immune response of the host. The immune response can help to eliminate the virus; however, the immune response may also cause liver damage. It has been established that tumor formation is strongly associated with host immune status, and tumors employ various mechanisms to evade immune surveillance. This is mainly mediated by inducing immune tolerance or immune-suppressive mechanisms. Among all immune cells, HBV-specific cluster of differentiation (CD) 8+ T cells play a critical role in viral clearance and the pathogenesis of HBV infection [71]. HBx promotes the apoptosis of CD8+ T lymphocytes and decreases the generation of interferon-γ; these actions attenuate the host immune response resulting in persistent HBV infection, which eventually contributes to the malignant transformation of hepatocytes [17]. In addition, HBx is also implicated in inflammation and immunomodulation during HBV infection through the upregulation of inflammatory cytokines and immune response-related molecules such as the major histocompatibility complex, ICAM-1, and Fas ligand [18, 19]. This suggests that HBx may contribute to liver inflammation through the upregulation of immune response-related molecules and that the consequent long-term chronic liver inflammation promotes HCC development. Conversely, HBx may also inhibit the HBV-specific immune response, modulate the host immune response to the tumor, promote apoptosis in immune cells, and induce immune tolerance. This eventually results in persistent HBV infection and a favorable tumor microenvironment for HCC initiation, progression, and invasion.

### Hepatitis B virus X protein and hepatic stellate cells

Hepatic stellate cells, also termed liver fat-storing cells, are mainly located between the hepatocytes and the sinusoidal space. Under normal circumstances, the main function of hepatic stellate cells is to store a small amount of vitamin A and to synthesize components of the extracellular matrix. In patients with liver injury, inflammatory and Kupffer cells secrete inflammatory cytokines that promote hepatic stellate cell activation. The activated hepatic stellate cells are then further transformed into myofibroblasts, which exhibit proliferation, migration, contraction, and protein synthesis. Hepatic stellate cells play an important role in the pathological processes of liver damage and fibrosis, and recent studies have indicated that they are associated with HCC invasion and metastasis [72]. As a major regulatory protein in the life cycle of HBV, HBx contributes to this pathological process by activating hepatic stellate cells. Recent studies investigating the mechanisms by which hepatic stellate cells are activated have focused on the TGF-β and platelet-derived growth factor (PDGF) signaling pathways, in which TGF-β is considered to play a key regulatory role. In vitro studies have shown that HBx upregulates TGF-β at both the protein translation and gene transcription levels. Furthermore, TGF-β activates Smads, which are downstream signaling molecules that are transported into the nucleus where they interact with DNA-binding proteins to exert their functions. HBx, involved in the TGF-β signaling pathway, contributes to the activation of hepatic stellate cells, promoting their proliferation and migration. Consequently, the activated hepatic stellate cells increase α-smooth muscle actin and matrix metalloproteinase (MMP) levels, promoting disorganization of the liver architecture and abnormal deposition of extracellular matrix collagen. This induces a series of pathological processes such as extracellular matrix remodeling, fibrosis, angiogenesis, HCC invasion, and metastasis [20, 21].

### Hepatitis B virus X protein and inflammatory cytokines

#### Hepatitis B virus X protein and transforming growth factor-β

Cytokines are a class of small molecule signaling proteins that exert their function by recognizing cell-surface receptors and facilitating communication between different cells. TGF-β, an inflammation-related cytokine belonging to the TGF-β superfamily, is mainly secreted by tumor cells, tumor-associated macrophages, and regulatory T cells in the tumor microenvironment. The dual role of TGF-β in cancer has long been recognized. TGF-β, exploited by cancer cells, is implicated in processes such as tumor invasion and tumor microenvironment regulation. In contrast, TGF-β also exerts tumor-suppressive effects. In other words, the output of the TGF-β signaling pathway depends on the stage of tumor development and type of tumor tissue [73]. Numerous studies have confirmed a close relationship between HBx and TGF-β. A previous study showed that HBx can switch the target of the intrahepatic TGF-β signaling pathway from the tumor-suppressive pSmad3C to the tumor-supportive pSmad3L during the early stages of chronic hepatitis B (CHB). This mechanism is considered to be directly involved in hepatocarcinogenesis [23]. HBx also functions by upregulating TGF-β in a paracrine-dependent manner. TGF-β is a master regulator of the pro-invasive tumor microenvironment. In addition to participating in hepatic stellate cell activation, TGF-β can also associate with pathways related to stem cell phenotypes such as Wnt and Ras signaling to induce EMT or can switch the phenotypes of tumor-infiltrating immune cells to create an EMT-permissive microenvironment. Accordingly, tumor cells undergoing EMT acquire invasive, migratory, and stem cell properties, which allows them to disseminate to distant sites [22, 24].

#### Hepatitis B virus X protein and the interleukin family

Interleukins, a type of cytokine with wide-ranging functions, are produced by various cells and play an important role in immune-cell maturation, activation, and regulation. During chronic liver inflammation, inflammatory cytokines are released, the activation of which contributes to the pathological process of chronic liver inflammation and injury. The balance of inflammatory cytokines will determine the final outcome of the immune response. Therefore, inflammatory cytokines are potential therapeutic targets for the treatment of liver disease. Reportedly, HBx regulates the expression of inflammatory cytokines at the transcription level, thus playing a key role in the regulation of chronic liver inflammation [74]. Previous studies have indicated that HBx activates NF-κB and MAPKs through the Toll-like receptor adaptor protein myeloid differentiation factor 88, thereby promoting the synthesis and secretion of IL-6 [25]. IL-1 is also upregulated by HBx at the transcription level. Levels of IL-6 and IL-1, the predominant pro-inflammatory cytokines involved in HCC development, are often higher in patients with HCC. Moreover, they have pleiotropic effects on various cell types in the tumor microenvironment; particularly, they are able to regulate the pro-oncogenic transcription factors NF-κB and STAT3. For this reason, such cytokines may influence key parameters of oncogenesis such as tumor invasion and metastasis, as well as the ability of tumor cells to respond to anti-cancer therapy [75, 76]. Therefore, HCC patients with high IL-6 levels typically have a poor prognosis [77]. In addition, HBx also selectively regulates other pro-inflammatory cytokines, including IL-8, IL-18, IL-23, and TNF-α [27]. These cytokines function in the pathological processes underlying HCC development. For instance, IL-8 regulates tumor growth and the malignant transformation of hepatocytes; additionally, it has been associated with HCC invasion and metastasis [26, 78]. Serum levels of IL-18, a novel pro-inflammatory cytokine, are often elevated in patients with HBV-HCC and may have an application as a prognostic indicator in these patients [79]. This evidence suggests that the cytokines upregulated by HBx are likely to be tumor-promoting chemokines active in HCC development and that the role of HBx in hepatocarcinogenesis may be effectuated by regulating inflammatory cytokine expression.

#### Hepatitis B virus X protein and tumor necrosis factor-α

As described above, long-term chronic liver inflammation mediates HCC initiation and development. Once liver inflammation is initiated, inflammatory cells are actively induced by tumor cells to infiltrate the liver tumor microenvironment, resulting in the release of inflammatory cytokines and corresponding chemokines [80]. As a classic pro-inflammatory cytokine, TNF-α is mainly secreted by inflammatory cells and regulates cell survival, proliferation, differentiation, and immune responses. Thus, it is recognized as one of the most important cytokines involved in HCC pathogenesis. Previous in vivo and in vitro studies have shown that TNF-α functions during tumor initiation and development [81]. The relationship between HBx and TNF-α was first reported in 1998. An in vitro HBV expression system showed that HBx upregulates the expression of TNF-α transcriptionally, after which it mediates liver inflammation and disease progression [28]. TNF-α can also upregulate the expression of vascular endothelial growth factor and MMPs and activate survival signaling pathways, thus promoting tumor development and angiogenesis [29]. This suggests that HBx may mediate liver inflammation, disease progression, and tumor survival via regulation of the expression of certain inflammatory cytokines. Further clarification of the mechanisms underlying the regulation of intrahepatic inflammatory cytokines may result in the discovery of promising future therapeutic targets.

#### Hepatitis B virus X protein and cyclooxygenase-2

Cyclooxygenase (COX) is the rate-limiting enzyme for arachidonic acid metabolism. Two subtypes have been identified to date, namely, COX-1 and COX-2. COX-2 has been characterized as a highly inducible isoform that is rapidly upregulated in response to proinflammatory triggers including cytokines, tissue injury, and mitogens, particularly in cells involved in inflammation, pain, and tumors. Previous studies have shown that COX-2 levels are often elevated in patients with CHB, cirrhosis, and HCC and that its expression is significantly correlated with that of HBx in the tumors of HBV-HCC patients. This suggests that COX-2 is a key factor in the contribution of HBx to the pathology of HCC. HBx upregulates the expression of MT1-MMP in a COX-2-dependent manner and exerts its anti-apoptotic effects by activating the COX-2/PGE(2) signaling pathway thus promoting tumor growth, invasion, and metastasis [30–32]. Moreover, colocalization of HBx with COX-3 may lead to the upregulation of COX-2, which promotes HepG2 cell growth [82]. Collectively, COX-2 activity is maintained by HBx in various ways, and HBx exerts its carcinogenic effects via this mechanism.

### Hepatitis B virus X protein and hypoxia-inducible factor-1α

The cellular oxygen balance is often impaired during cancer, and cells become hypoxic. Hypoxia is a common feature in many types of solid tumor such as liver cancer, where tumor cells rapidly proliferate, eventually forming large solid tumor masses. Consequently, aberrant blood vessels are generated around the tumor masses. To survive in this hypoxic microenvironment, tumor cells adapt to low oxygen conditions by activating a series of survival pathways, among which activation of hypoxia-inducible factor-1α (HIF-1α) is the most recognized. Many studies have shown that HIF-1α expression is increased in various tumors in humans including bladder, breast, and liver tumors [83, 84], and compelling evidence indicates a strong correlation between elevated HIF-1α levels and tumor invasion, angiogenesis, and poor patient prognosis [85, 86]. The role of HBx in HIF-1α expression and function has been investigated. Reportedly, HBx increases HIF-1α levels in two ways. First, HBx directly binds to the basic helix-loop-helix/PAS domain of HIF-1α to inhibit the interaction between pVHL and HIF-1α, thus preventing HIF-1α degradation [33, 34]. Second, HBx stimulates metastasis-associated protein 1, histone deacetylase, and the MAPK pathway [35] to upregulate HIF-1α expression. Therefore, HBx may facilitate the adaptation of tumor cells to low oxygen conditions through HIF-1α upregulation, allowing tumor cells to survive in this harsh microenvironment.

### Hepatitis B virus X protein and exosomes

Exosomes are small (50–150 nm) membrane vesicles with diverse functions that are released by various cells, including hepatocytes. Cancer cell-derived exosomes have a vast array of contents comprising miRNAs, mRNAs, transcription factors, proteins, and lipids. The contents of exosomes are functional and exert powerful effects on recipient cells. One of the most distinguishing hallmarks of cancer cell-derived exosomes is their high miRNA content. Exosomal miRNAs can regulate various physiological cellular events. As the pivotal mediators of communication in the tumor microenvironment, exosome-mediated cell–cell communication can alter tumor growth, migration, and metastasis. In recent years, most studies have focused on the role of HBx in exosomal miRNA expression and function. Mounting evidence suggests that HBx affects cancer cell proliferation, apoptosis, and migration through the regulation of miRNA expression. miR-122 is a liver-specific miRNA present in exosomes. According to previous studies, miR-122 represses HCC development by binding to the target genes involved in proliferation, migration, differentiation, apoptosis, and angiogenesis in HCC. Decreased miR-122 expression is frequently observed in patients with HCC [87, 88]. Reportedly, HBx exerts its carcinogenic effects through the negative regulation of miR-122 expression [36]. Recently, Zhao et al. [37] found that HBx also significantly alters exosomal protein content in vitro. This suggests that HBx exerts powerful effects via the exosome by regulating both exosomal miRNA and protein content. Therefore, HBx-induced exosome content changes may help to create an intracellular environment favorable for virus-associated malignant transformation as well as tumor invasion and metastasis. Further clarification of the role of HBx in exosome regulation and function will contribute to our understanding of the molecular basis of HBV-HCC pathology.

## Potential uses of hepatitis B virus X protein in hepatocellular carcinoma treatment

HBx has become an important biological indicator for HBV-HCC development. HBx activity and function in the tumor microenvironment may become a new therapeutic target for the treatment of HCC. Knockdown of HBx expression using short interfering or short hairpin RNAs or inhibitions of the signaling pathways that are activated by HBx reportedly abrogate the functions of HBx [69, 89]. To date, most studies have utilized in vitro experiments and in vivo experiments in animal models, and research on humans has not been performed; therefore, strategies targeting HBx in human tumors require further investigation.

## Conclusions and future perspectives

**Fig. 3:**
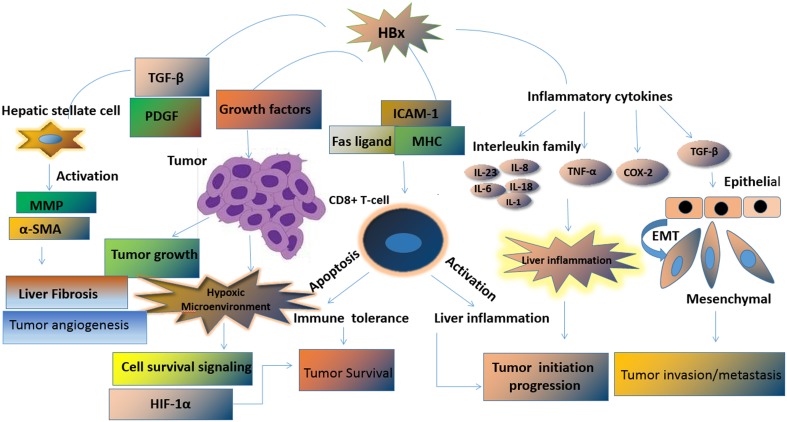
Pleiotropic effects of HBx in liver tumor microenvironment

HBx is a multifunctional viral protein encoded by HBV. In recent decades, much has been discovered about the role of HBx in the initiation, progression, invasion, and metastasis of HCC. HBx plays an important role in HBV-associated liver diseases by activating a series of intracellular signaling pathways that are involved in the progression from chronic hepatitis to cirrhosis and eventually HCC. The various biological functions of HBx are exerted at different intracellular locations. HBx activates hepatic stellate cells, accelerates tumor cell growth, dysregulates the anti-tumor immune response, promotes liver inflammation, and induces EMT in tumor cells. The biological effects of HBx are mainly mediated by its interaction with constituents in the liver tumor microenvironments such as liver cancer cells, hepatic stellate cells, immune cells, inflammatory cytokines, HIF-1α, and exosomes. Complex interactions between HBx and components of the tumor microenvironment ultimately promote tumor initiation, progression, invasion, and metastasis (Fig. 3). Improving our understanding of the relationship between HBx and the tumor microenvironment is critical for the identification of diagnostic biomarkers and to design novel therapeutic approaches. However, some questions remain to be answered. First, which domains and host targets are responsible for the pleiotropic effects of HBx? Second, how does HBx control the level of HBV replication and exert its cofactor role in HCC? Third, how can the biological effects of HBx be effectively abrogated? Answers to these questions would help identify the promising therapeutic targets and thus improve the outcomes of HBV-HCC patients.

## Acknowledgments

This work was supported by grants from Special National International Technology Cooperation of China (No. OS2015ZR1028/2015DFA31490), National Natural Sciences Foundation of China (Nos. 81272253 and 81201619 ), and National Major Sciences Research Program of China (973 Program) (No. 2013CB910502).

## References

[1] Ferlay J, Shin HR, Bray F, Forman D, Mathers C, Parkin DM (2010). Estimates of worldwide burden of cancer in 2008: GLOBOCAN 2008. Int J Cancer.

[2] Beasley RP, Hwang LY, Lin CC, Chien CS (1981). Hepatocellular carcinoma and hepatitis B virus. A prospective study of 22 707 men in Taiwan. Lancet.

[3] Jiang Z, Jhunjhunwala S, Liu J, Haverty PM, Kennemer MI, Guan Y, Lee W, Carnevali P, Stinson J, Johnson S, Diao J, Yeung S, Jubb A, Ye W, Wu TD, Kapadia SB, de Sauvage FJ, Gentleman RC, Stern HM, Seshagiri S, Pant KP, Modrusan Z, Ballinger DG, Zhang Z (2012). The effects of hepatitis B virus integration into the genomes of hepatocellular carcinoma patients. Genome Res.

[4] Clevers H (2004). At the crossroads of inflammation and cancer. Cell.

[5] Benn J, Schneider RJ (1995). Hepatitis B virus HBx protein deregulates cell cycle checkpoint controls. Proc Natl Acad Sci U S A.

[6] Liu H, Yuan Y, Guo H, Mitchelson K, Zhang K, Xie L, Qin W, Lu Y, Wang J, Guo Y, Zhou Y, He F (2012). Hepatitis B virus encoded X protein suppresses apoptosis by inhibition of the caspase-independent pathway. J Proteome Res.

[7] Cho HK, Kim SY, Kyaw YY, Win AA, Koo SH, Kim HH, Cheong J (2015). HBx induces the proliferation of hepatocellular carcinoma cells via AP1 over-expressed as a result of ER stress. Biochem J.

[8] Neviani P, Fabbri M (2015). Exosomic microRNAs in the tumor microenvironment. Front Med (Lausanne).

[9] Nagaraju GP, Bramhachari PV, Raghu G, El-Rayes BF (2015). Hypoxia inducible factor-1alpha: Its role in colorectal carcinogenesis and metastasis. Cancer Lett.

[10] Kise K, Kinugasa-Katayama Y, Takakura N (2016). Tumor microenvironment for cancer stem cells. Adv Drug Deliv Rev.

[11] Capece D, Fischietti M, Verzella D, Gaggiano A, Cicciarelli G, Tessitore A, Zazzeroni F, Alesse E (2013). The inflammatory microenvironment in hepatocellular carcinoma: A pivotal role for tumor-associated macrophages. Biomed Res Int.

[12] Guo L, Zhang Y, Zhang L, Huang F, Li J, Wang S (2016). MicroRNAs, TGF-beta signaling, and the inflammatory microenvironment in cancer. Tumour Biol.

[13] Balkwill F, Mantovani A (2001). Inflammation and cancer: back to Virchow?. Lancet.

[14] Mantovani A, Allavena P, Sica A, Balkwill F (2008). Cancer-related inflammation. Nature.

[15] Hanahan D, Weinberg RA (2011). Hallmarks of cancer: the next generation. Cell.

[16] Zhang HT, Chen GG, Hu BG, Zhang ZY, Yun JP, He ML, Lai PB (2014). Hepatitis B virus x protein induces autophagy via activating death-associated protein kinase. J Viral Hepat.

[17] Lee MJ, Jin YH, Kim K, Choi Y, Kim HC, Park S (2010). Expression of hepatitis B virus x protein in hepatocytes suppresses CD8 T cell activity. Immune Netw.

[18] Zhou DX, Taraboulos A, Ou JH, Yen TS (1990). Activation of class I major histocompatibility complex gene expression by hepatitis B virus. J Virol.

[19] Kim SY, Kim JK, Kim HJ, Ahn JK (2005). Hepatitis B virus X protein sensitizes UV-induced apoptosis by transcriptional transactivation of Fas ligand gene expression. IUBMB LIFE.

[20] Martin-Vilchez S, Sanz-Cameno P, Rodriguez-Munoz Y, Majano PL, Molina-Jimenez F, Lopez-Cabrera M, Moreno-Otero R, Lara-Pezzi E (2008). The hepatitis B virus X protein induces paracrine activation of human hepatic stellate cells. Hepatology.

[21] Bai Q, An J, Wu X, You H, Ma H, Liu T, Gao N, Jia J (2012). HBV promotes the proliferation of hepatic stellate cells via the PDGF-B/PDGFR-beta signaling pathway in vitro. Int J Mol Med.

[22] Chen HY, Chen ZX, Huang RF, Lin N (2014). Hepatitis B virus X protein activates human hepatic stellate cells through upregulating TGFβ1. Genet Mol Res.

[23] Murata M, Matsuzaki K, Yoshida K, Sekimoto G, Tahashi Y, Mori S, Uemura Y, Sakaida N, Fujisawa J, Seki T, Kobayashi K, Yokote K, Koike K, Okazaki K (2009). Hepatitis B virus X protein shifts human hepatic transforming growth factor (TGF)-beta signaling from tumor suppression to oncogenesis in early chronic hepatitis B. Hepatology.

[24] Scheel C, Eaton EN, Li SH, Chaffer CL, Reinhardt F, Kah KJ, Bell G, Guo W, Rubin J, Richardson AL, Weinberg RA (2011). Paracrine and autocrine signals induce and maintain mesenchymal and stem cell states in the breast. Cell.

[25] Xiang WQ, Feng WF, Ke W, Sun Z, Chen Z, Liu W (2011). Hepatitis B virus X protein stimulates IL-6 expression in hepatocytes via a MyD88-dependent pathway. J Hepatol.

[26] Wang D, Zou L, Liu X, Zhu H, Zhu R (2016). Chemokine expression profiles of human hepatoma cell lines mediated by hepatitis b virus x protein. Pathol Oncol Res : POR.

[27] Xia L, Tian D, Huang W, Zhu H, Wang J, Zhang Y, Hu H, Nie Y, Fan D, Wu K (2012). Upregulation of IL-23 expression in patients with chronic hepatitis B is mediated by the HBx/ERK/NF-kB pathway. J Immunol.

[28] Lara-Pezzi E, Majano PL, Gomez-Gonzalo M, Garcia-Monzon C, Moreno-Otero R, Levrero M, Lopez-Cabrera M (1998). The hepatitis B virus X protein up-regulates tumor necrosis factor alpha gene expression in hepatocytes. Hepatology.

[29] Van Horssen R, Ten HT, Eggermont AM (2006). TNF-alpha in cancer treatment: Molecular insights, antitumor effects, and clinical utility. Oncologist.

[30] Lara-Pezzi E, Gomez-Gaviro MV, Galvez BG, Mira E, Iniguez MA, Fresno M, Martinez-A C, Arroyo AG, Lopez-Cabrera M (2002). The hepatitis B virus X protein promotes tumor cell invasion by inducing membrane-type matrix metalloproteinase-1 and cyclooxygenase-2 expression. J Clin Invest.

[31] Cheng AS, Yu J, Lai PB, Chan HL, Sung JJ (2008). COX-2 mediates hepatitis B virus X protein abrogation of p53-induced apoptosis. Biochem Biophys Res Commun.

[32] Liu KG, Shao XL, Xie HH, Xu L, Zhao H, Guo ZH, Li L, Liu J (2010). [the expression of hepatitis B virus X protein and cyclooxygenase-2 in hepatitis B virus-related hepatocellular carcinoma: correlation with microangiogenesis and metastasis, and what is the possible mechanism]. Zhonghua Gan Zang Bing Za Zhi.

[33] Yoo YG, Oh SH, Park ES, Cho H, Lee N, Park H, Kim DK, Yu DY, Seong JK, Lee MO (2003). Hepatitis B virus X protein enhances transcriptional activity of hypoxia-inducible factor-1alpha through activation of mitogen-activated protein kinase pathway. J Biol Chem.

[34] Yoo YG, Lee MO (2004). Hepatitis B virus X protein induces expression of Fas ligand gene through enhancing transcriptional activity of early growth response factor. J Biol Chem.

[35] Yoo YG, Na TY, Seo HW, Seong JK, Park CK, Shin YK, Lee MO (2008). Hepatitis B virus X protein induces the expression of MTA1 and HDAC1, which enhances hypoxia signaling in hepatocellular carcinoma cells. Oncogene.

[36] Song K, Han C, Zhang J, Lu D, Dash S, Feitelson M, Lim K, Wu T (2013). Epigenetic regulation of MicroRNA-122 by peroxisome proliferator activated receptor-gamma and hepatitis b virus X protein in hepatocellular carcinoma cells. Hepatology.

[37] Zhao X, Wu Y, Duan J, Ma Y, Shen Z, Wei L, Cui X, Zhang J, Xie Y, Liu J (2014). Quantitative proteomic analysis of exosome protein content changes induced by hepatitis B virus in Huh-7 cells using SILAC labeling and LC-MS/MS. J Proteome Res.

[38] Vogelstein B, Kinzler KW (2004). Cancer genes and the pathways they control. Nat Med.

[39] Friedman SL (2008). Mechanisms of hepatic fibrogenesis. Gastroenterology.

[40] Amann T, Bataille F, Spruss T, Muehlbauer M, Gaebele E, Schoelmerich J, Kiefer P, Bosserhoff A, Hellerbrand C (2009). Activated hepatic stellate cells promote tumorigenicity of hepatocellular carcinoma. Cancer Sci.

[41] Wong VW, Yu J, Cheng AS, Wong GL, Chan HY, Chu ES, Ng EK, Chan FK, Sung JJ, Chan HL (2009). High serum interleukin-6 level predicts future hepatocellular carcinoma development in patients with chronic hepatitis B. Int J Cancer.

[42] Huang YS, Hwang SJ, Chan CY, Wu JC, Chao Y, Chang FY, Lee SD (1999). Serum levels of cytokines in hepatitis C-related liver disease: a longitudinal study. Zhonghua yi xue za zhi = Chin Med J; Free China ed.

[43] Kakumu S, Okumura A, Ishikawa T, Yano M, Enomoto A, Nishimura H, Yoshioka K, Yoshika Y (1997). Serum levels of IL-10, IL-15 and soluble tumour necrosis factor-alpha (TNF-alpha) receptors in type C chronic liver disease. Clin Exp Immunol.

[44] Massague J (2008). TGFbeta in cancer. Cell.

[45] Unitt E, Marshall A, Gelson W, Rushbrook SM, Davies S, Vowler SL, Morris LS, Coleman N, Alexander GJ (2006). Tumour lymphocytic infiltrate and recurrence of hepatocellular carcinoma following liver transplantation. J Hepatol.

[46] Buonaguro L, Petrizzo A, Tagliamonte M, Tornesello ML, Buonaguro FM (2013). Challenges in cancer vaccine development for hepatocellular carcinoma. J Hepatol.

[47] Kidd-Ljunggren K, Oberg M, Kidd AH (1995). The hepatitis B virus X gene: Analysis of functional domain variation and gene phylogeny using multiple sequences. J Gen Virol.

[48] Cheong JH, Yi M, Lin Y, Murakami S (1995). Human RPB5, a subunit shared by eukaryotic nuclear RNA polymerases, binds human hepatitis B virus X protein and may play a role in X transactivation. EMBO J.

[49] Haviv I, Shamay M, Doitsh G, Shaul Y (1998). Hepatitis B virus pX targets TFIIB in transcription coactivation. Mol Cell Biol.

[50] Lin Y, Nomura T, Cheong J, Dorjsuren D, Iida K, Murakami S (1997). Hepatitis B virus X protein is a transcriptional modulator that communicates with transcription factor IIB and the RNA polymerase II subunit 5. J Biol Chem.

[51] Tiollais P, Charnay P, Vyas GN (1981). Biology of hepatitis B virus. Science.

[52] Wang HD, Trivedi A, Johnson DL (1997). Hepatitis B virus X protein induces RNA polymerase III-dependent gene transcription and increases cellular TATA-binding protein by activating the Ras signaling pathway. Mol Cell Biol.

[53] Wang HD, Trivedi A, Johnson DL (1998). Regulation of RNA polymerase I-dependent promoters by the hepatitis B virus X protein via activated Ras and TATA-binding protein. Mol Cell Biol.

[54] Lu B, Guo H, Zhao J, Wang C, Wu G, Pang M, Tong X, Bu F, Liang A, Hou S, Fan X, Dai J, Wang H, Guo Y (2010). Increased expression of iASPP, regulated by hepatitis B virus X protein-mediated NF-kappaB activation, in hepatocellular carcinoma. Gastroenterology.

[55] Cha MY, Kim CM, Park YM, Ryu WS (2004). Hepatitis B virus X protein is essential for the activation of Wnt/beta-catenin signaling in hepatoma cells. Hepatology.

[56] Arbuthnot P, Capovilla A, Kew M (2000). Putative role of hepatitis B virus X protein in hepatocarcinogenesis: effects on apoptosis, DNA repair, mitogen-activated protein kinase and JAK/STAT pathways. J Gastroenterol Hepatol.

[57] Chin R, Earnest-Silveira L, Koeberlein B, Franz S, Zentgraf H, Dong X, Gowans E, Bock CT, Torresi J (2007). Modulation of MAPK pathways and cell cycle by replicating hepatitis B virus: factors contributing to hepatocarcinogenesis. J Hepatol.

[58] Cheng B, Zheng Y, Guo X, Wang Y, Liu C (2010). Hepatitis B viral X protein alters the biological features and expressions of DNA repair enzymes in LO2 cells. Liver Int.

[59] Lee YI, Hwang JM, Im JH, Lee YI, Kim NS, Kim DG, Yu DY, Moon HB, Park SK (2004). Human hepatitis B virus-X protein alters mitochondrial function and physiology in human liver cells. J Biol Chem.

[60] Takada S, Shirakata Y, Kaneniwa N, Koike K (1999). Association of hepatitis B virus X protein with mitochondria causes mitochondrial aggregation at the nuclear periphery, leading to cell death. Oncogene.

[61] Cho HK, Cheong KJ, Kim HY, Cheong J (2011). Endoplasmic reticulum stress induced by hepatitis B virus X protein enhances cyclo-oxygenase 2 expression via activating transcription factor 4. Biochem J.

[62] Huang J, Kwong J, Sun EC, Liang TJ (1996). Proteasome complex as a potential cellular target of hepatitis B virus X protein. J Virol.

[63] Sirma H, Weil R, Rosmorduc O, Urban S, Israel A, Kremsdorf D, Brechot C (1998). Cytosol is the prime compartment of hepatitis B virus X protein where it colocalizes with the proteasome. Oncogene.

[64] Tanaka Y, Kanai F, Kawakami T, Tateishi K, Ijichi H, Kawabe T, Arakawa Y, Kawakami T, Nishimura T, Shirakata Y, Koike K, Omata M (2004). Interaction of the hepatitis B virus X protein (HBx) with heat shock protein 60 enhances HBx-mediated apoptosis. Biochem Biophys Res Commun.

[65] Wei Y, Neuveut C, Tiollais P, Buendia MA (2010). Molecular biology of the hepatitis B virus and role of the X gene. Pathol Biol (Paris).

[66] Wang D, Cai H, Yu WB, Yu L (2014). Identification of hepatitis B virus X gene variants between hepatocellular carcinoma tissues and pericarcinoma liver tissues in Eastern China. Int J Clin Exp Pathol.

[67] Wang Q, Zhang WY, Ye LH, Zhang XD (2010). A mutant of HBx (HBxDelta127) promotes hepatoma cell growth via sterol regulatory element binding protein 1c involving 5-lipoxygenase. Acta Pharmacol Sin.

[68] Zhang H, Shan CL, Li N, Zhang X, Zhang XZ, Xu FQ, Zhang S, Qiu LY, Ye LH, Zhang XD (2008). Identification of a natural mutant of HBV X protein truncated 27 amino acids at the COOH terminal and its effect on liver cell proliferation. Acta Pharmacol Sin.

[69] Wu YH, Ai X, Liu FY, Liang HF, Zhang BX, Chen XP (2016). C-Jun N-terminal kinase inhibitor favors transforming growth factor-beta to antagonize hepatitis B virus X protein-induced cell growth promotion in hepatocellular carcinoma. Mol Med Rep.

[70] Arzumanyan A, Friedman T, Ng IO, Clayton MM, Lian Z, Feitelson MA (2011). Does the hepatitis B antigen HBx promote the appearance of liver cancer stem cells?. Cancer Res.

[71] Thimme R, Wieland S, Steiger C, Ghrayeb J, Reimann KA, Purcell RH, Chisari FV (2003). CD8(+) T cells mediate viral clearance and disease pathogenesis during acute hepatitis B virus infection. J Virol.

[72] Vidal-Vanaclocha F (2008). The prometastatic microenvironment of the liver. Cancer Microenviron.

[73] Wang C, Wang C, Wei Z, Li Y, Wang W, Li X, Zhao J, Zhou X, Qu X, Xiang F (2015). Suppression of motor protein KIF3C expression inhibits tumor growth and metastasis in breast cancer by inhibiting TGF-beta signaling. Cancer Lett.

[74] Almajhdi FN, Al-Qudari AY, Hussain Z (2013). Differential expression of transforming growth factor-beta1 and HBx enhances hepatitis B virus replication and augments host immune cytokines and chemokines. Ann Hepatol.

[75] Tawara K, Oxford JT, Jorcyk CL (2011). Clinical significance of interleukin (IL)-6 in cancer metastasis to bone: potential of anti-IL-6 therapies. Cancer Manag Res.

[76] Chang Q, Bournazou E, Sansone P, Berishaj M, Gao SP, Daly L, Wels J, Theilen T, Granitto S, Zhang X, Cotari J, Alpaugh ML, de Stanchina E, Manova K, Li M, Bonafe M, Ceccarelli C, Taffurelli M, Santini D, Altan-Bonnet G, Kaplan R, Norton L, Nishimoto N, Huszar D, Lyden D, Bromberg J (2013). The IL-6/JAK/Stat3 feed-forward loop drives tumorigenesis and metastasis. Neoplasia.

[77] Sheng T, Wang B, SY W, Deng B, Qu L, Qi XS, Wang XL, Deng GL, Sun X (2015). The relationship between serum interleukin-6 and the recurrence of hepatitis b virus related hepatocellular carcinoma after curative resection. Medicine (Baltimore).

[78] Li XP, Yang XY, Biskup E, Zhou J, Li HL, Wu YF, Chen ML, Xu F (2015). Coexpression of CXCL8 and HIF-1αis associated with metastasis and poor prognosis in hepatocellular carcinoma. Oncotarget.

[79] Tangkijvanich P, Thong-Ngam D, Mahachai V, Theamboonlers A, Poovorawan Y (2007). Role of serum interleukin-18 as a prognostic factor in patients with hepatocellular carcinoma. World J Gastroenterol.

[80] Brigati C, Noonan DM, Albini A, Benelli R (2002). Tumors and inflammatory infiltrates: Friends or foes?. Clin Exp Metastasis.

[81] Moore RJ, Owens DM, Stamp G, Arnott C, Burke F, East N, Holdsworth H, Turner L, Rollins B, Pasparakis M, Kollias G, Balkwill F (1999). Mice deficient in tumor necrosis factor-alpha are resistant to skin carcinogenesis. Nat Med.

[82] Zheng BY, Fang XF, Zou LY, Huang YH, Chen ZX, Li D, Zhou LY, Chen H, Wang XZ (2014). The co-localization of HBx and COXIII upregulates COX-2 promoting HepG2 cell growth. Int J Oncol.

[83] Harris AL (2002). Hypoxia--a key regulatory factor in tumour growth. Nat Rev Cancer.

[84] Semenza GL (2003). Targeting HIF-1 for cancer therapy. Nat Rev Cancer.

[85] Lin D, Wu J (2015). Hypoxia inducible factor in hepatocellular carcinoma: A therapeutic target. World J Gastroenterol.

[86] Liu ZJ, Semenza GL, Zhang HF (2015). Hypoxia-inducible factor 1 and breast cancer metastasis. J Zhejiang Univ Sci B.

[87] Hu J, Xu Y, Hao J, Wang S, Li C, Meng S (2012). MiR-122 in hepatic function and liver diseases. Protein Cell.

[88] Szabo G, Bala S (2013). MicroRNAs in liver disease. Nat Rev Gastroenterol Hepatol.

[89] Chan DW, Ng IO (2006). Knock-down of hepatitis B virus X protein reduces the tumorigenicity of hepatocellular carcinoma cells. J Pathol.

